# Effects of *Bifidobacterium* on metabolic parameters in overweight or obesity adults: a systematic review and meta-analysis

**DOI:** 10.3389/fmicb.2025.1633434

**Published:** 2025-09-25

**Authors:** Junmei Huang, Hao Cheng

**Affiliations:** Department of Nutrition, Sanya Central Hospital (The Third People's Hospital of Hainan Province), Sanya, China

**Keywords:** *Bifidobacteria*, overweight or obesity, weight, blood glucose, blood lipids

## Abstract

**Objective:**

This study endeavors to elucidate how *Bifidobacteria* supplementation affects metabolic parameters among overweight or obese populations.

**Methods:**

A systematic review and meta-analysis were carried out leveraging PubMed, Embase, Cochrane Library, and Web of Science. Merely randomized controlled trials (RCTs) were included.

**Results:**

21 RCTs were encompassed for our final analysis. *Bifidobacteria* supplementation was effective in weight management for overweight or obese patients. The experimental group receiving *Bifidobacteria* exhibited a marked decrease in weight (WMD: −0.607 kg; 95% CI: −0.910, −0.303, *I^2^* = 11.9%) and BMI (WMD: −0.214 kg/m^2^; 95% CI: −0.259, −0.169, *I^2^* = 4.1%) in contrast to the control, although the significant effect was not noted on WC. Moreover, while *Bifidobacteria* supplementation led to no marked drop in FBG or HbA1c, it improved the insulin (SMD: -0.268; 95% CI: −0.470, −0.066, *I^2^* = 5.4%). However, there were no evident variations in TC, TG, HDL-C, or LDL-C across groups.

**Conclusion:**

Our study findings confirmed that *Bifidobacteria* contributes to a slight reduction in weight and BMI among the overweight or obese populations, making it a potential adjunctive approach for weight management. Furthermore, it may help regulate insulin levels, though its impact on hyperglycemia and hyperlipidemia remains limited.

**Systematic review registration:**

https://www.crd.york.ac.uk/PROSPERO. Registration No. CRD42025635324.

## 1 Introduction

As the global obesity epidemic continues to intensify, overweight and obesity have become significant public health concerns worldwide (GBD 2021 Adult BMI Collaborators, 2025). Obesity not only diminishes an individual’s quality of life but is also strongly associated with various chronic diseases, such as cardiovascular diseases, type 2 diabetes mellitus (T2DM), certain cancers, and a range of metabolic disorders (Blüher, 2019). Consequently, identifying effective interventions for the prevention and treatment of obesity has become increasingly critical.

In recent, growing attention has been directed towards the relationship between the gut microbiota and obesity (Asadi et al., 2022). The gut microbiota, an intricate ecosystem involving trillions of microorganisms, is crucial in host metabolism, immunity, as well as disease susceptibility (Geng et al., 2022). Among these microorganisms, *Bifidobacteria*, an important component of the gut microbiota, has garnered particular attention due to its potential health benefits (Sarita et al., 2024). *Bifidobacteria* are Gram-positive, anaerobic probiotics known to exert multiple positive effects on host health, including improving gut barrier function, modulating immune responses, and influencing energy metabolism (Amabebe et al., 2020). Multiple studies have demonstrated that the abundance of *Bifidobacterium* in the gut of obese individuals is generally lower than that in healthy populations (Leite et al., 2024; Gong et al., 2022). This dysbiosis may be associated with high-fat diets, metabolic disturbances, and chronic inflammation (Hills et al., 2019). Experimental evidence indicates that reduced *Bifidobacterium* abundance, accompanied by fat accumulation and inflammation, can be ameliorated by supplementation with specific *Bifidobacterium* strains, leading to attenuated weight gain and reduced fat deposition in obese mice (Zha et al., 2024). Furthermore, Da Silva et al. (2020) found that the proportion of *Bifidobacterium* in the gut microbiota of obese children was significantly lower than that of healthy counterparts. Some studies have suggested that the abundance of *Bifidobacterium* may be restored following bariatric surgery or dietary interventions (Seganfredo et al., 2017). The potential mechanisms may involve metabolic regulation mediated by short-chain fatty acids (SCFAs). For example, acetate and propionate can activate G-protein-coupled receptors (GPR41/GPR43), thereby suppressing lipogenesis and promoting energy utilization (Yun et al., 2024). Butyrate may stimulate the secretion of GLP-1 and PYY, reducing appetite through hormonal regulation (Horiuchi et al., 2020). In addition, SCFAs can enhance AMP-activated protein kinase (AMPK) activity, thereby facilitating fatty acid *β*-oxidation and promoting fat catabolism. *Bifidobacterium* may also influence bile acid metabolism by activating farnesoid X receptor (FXR) and TGR5, thereby improving glucose and lipid metabolism (Joyce and Gahan, 2016). Although studies have suggested that *Bifidobacteria* may be closely linked to weight management and glucose and lipid metabolic homeostasis in the population with excess weight or obesity, related findings show discrepancies, and a systematic evaluation and quantitative analysis are lacking. For instance, Bai et al. (2024) proved that the short-chain *Bifidobacterium* BBr60 markedly lowered weight, body mass index (BMI), and FBG, and modulated lipid profiles safely and effectively. In contrast, Sudha et al. (2019) found no significant changes in blood lipids or blood glucose levels. Additionally, some studies have indicated that yogurt containing *Bifidobacteria* does not produce significant changes in weight or BMI in overweight or obese populations.

However, the evidence remains inconsistent. Some trials have demonstrated beneficial effects of *Bifidobacteria* supplementation on weight, glucose, and lipid metabolism, while other studies report negligible or no benefits. To date, no comprehensive quantitative synthesis has been performed to resolve these discrepancies. Therefore, this study seeks to unveil the effects of *Bifidobacteria* supplementation on metabolic parameters within individuals with excess weight or obesity based on randomized controlled trials (RCTs) via systematic review and meta-analysis.

## 2 Methods

### 2.1 Registration and PRISMA statement

This review complied with the Preferred Reporting Items for Systematic Reviews and Meta-Analyses (PRISMA) guidelines and was prospectively registered in PROSPERO (Registration No. CRD42025635324) (Page et al., 2021).

### 2.2 Literature search

#### 2.2.1 Data sources and search scope

A comprehensive literature search was performed in four online databases (PubMed, Embase, Cochrane Library, and Web of Science) to identify RCTs. The search covered the period from database inception to December 3, 2024. Keywords such as “*Bifidobacterium*,” “obesity,” and “overweight” were used as both subject terms and free words in the search. The search strategy is presented in Table 1. The references of prior systematic reviews were also checked for eligible RCTs.

**Table 1:** Search strategy and results.

Pubmed		
#	Query	Results
1	Overweight[MeSH Terms]	289,803
2	Obesity[MeSH Terms]	277,956
3	‘adipose tissue hyperplasia’[Title/Abstract] OR ‘adipositas’[Title/Abstract] OR ‘adiposity’[Title/Abstract] OR ‘alimentary obesity’[Title/Abstract] OR ‘body weight, excess’[Title/Abstract] OR ‘corpulency’[Title/Abstract] OR ‘fat overload syndrome’[Title/Abstract] OR ‘nutritional obesity’[Title/Abstract] OR ‘obesitas’[Title/Abstract] OR ‘obesity’[Title/Abstract] OR ‘overweight’[Title/Abstract]	406,017
4	Bifidobacterium[MeSH Terms]	7,796
5	Bifidobacterium[Title/Abstract]	14,126
6	(#1 OR #2 OR #3) AND (#4 OR #5)	873

#### 2.2.2 Eligibility criteria

Inclusion criteria: (1) Adults (≥18) diagnosed by a physician with overweight (BMI 25–29.9 kg/m^2^) or obesity (BMI 30–34.9 kg/m^2^); (2) Explicit use of Bifidobacterium as an intervention; (3) A control group receiving either a placebo or another type of intervention not involving *Bifidobacterium*, with all other conditions equivalent to the experimental group, ensuring comparability (e.g., routine care or blank control); (4) Outcome measures including anthropometric measurements, blood glucose, or lipid laboratory result; (5) Randomized controlled trials (RCTs). Exclusion criteria: (1) Duplicates, systematic reviews, and meta-analyses; (2) Literature reviews, case reports, non-English publications, animal studies, letters to the editor, and narrative reviews; (3) Full texts were inaccessible, data could not be extracted, or the studies were ineligible.

#### 2.2.3 Literature screening and data extraction

Two researchers independently filtered the literature, collected related data, and assessed study quality. EndNote was utilized during the screening process. The extracted data encompassed bibliographic information (authors, publication year, country), study characteristics (sample size, bacterial strains, study outcomes), and participant characteristics (age, BMI). Data extraction was conducted utilizing Excel. Dissents were addressed via discussion or consultation with a third researcher.

#### 2.2.4 Outcome measures

The primary outcomes assessed in this meta-analysis were: weight, BMI, as well as waist circumference (WC). Secondary outcomes encompassed fasting blood glucose (FBG), glycated hemoglobin (HbA1c), insulin, total cholesterol (TC), triglycerides (TG), high-density lipoprotein cholesterol (HDL-C), and low-density lipoprotein cholesterol (LDL-C).

### 2.3 Risk of bias assessment

The risk of bias was rated independently by two reviewers. Dissents were addressed after discussion or judgment of a third party. Concerning quality assessment, both reviewers used the NIH RCT Quality Assessment Tool for study quality evaluation. There were 14 items, each assessed as “Yes” or “No” based on the study’s design and implementation standards (NIH, n.d.). In case of discrepancies during the evaluation, a third researcher was consulted to ensure fairness and consistency. Studies were rated for potential bias via a scoring scale, with three distinct categories: high risk (0–5, poor), moderate risk (6–10, fair), and low risk (11–14, good).

### 2.4 Statistical analysis

Data analysis was enabled by Stata MP 15. Continuous data were shown in standardized mean difference (SMD) or mean difference (MD). An SMD of < 0.2 indicates a negligible difference between groups, whereas a value ranging from 0.2 to 0.49 suggests a small difference. A value between 0.5 and 0.79 represents a moderate difference, and a value ≥ 0.8 denotes a substantial difference across groups. For dichotomous data, the risk ratio (RR) and its 95% confidence interval (CI) were computed. Heterogeneity was detected via the *I^2^* statistic and the Q-test. When the *I^2^* value exceeded 50% and the *p* < 0.05, a random-effects model was applied; otherwise, a fixed-effects model was used. The robustness of our results was rated through sensitivity analyses. For meta-analyses involving over 10 studies, possible publication bias was examined utilizing funnel plots and Egger’s test. In cases of bias, the effect on the results was evaluated through the trim-and-fill approach.

## 3 Results

### 3.1 Literature search results

**Figure 1:**
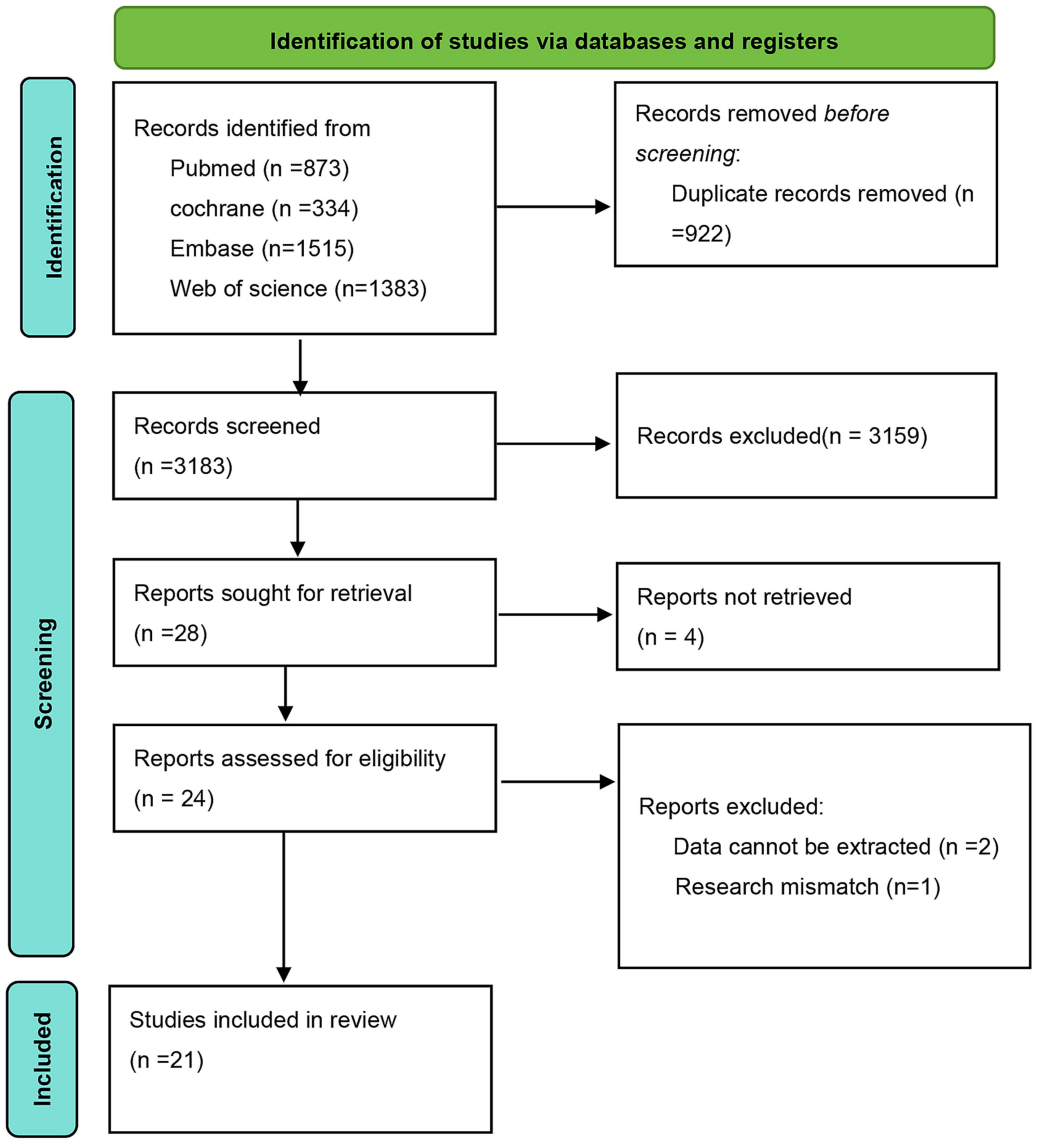
Study screening flowchart.

Based on the predefined search strategy, a preliminary search of the databases identified 4,105 articles. After screening titles and abstracts, 922 duplicate entries were excluded, resulting in 3,159 articles being discarded. The remaining 28 articles were subjected to full-text review. Four articles were excluded owing to unavailable full texts, two owing to the inability to extract data, and one due to failure to meet the inclusion criteria. Ultimately, 21 eligible RCTs were included, as shown in Figure 1.

### 3.2 Study characteristics

The baseline characteristics of the 21 included RCTs are summarized in Table 2. Of these, 13 (Bai et al., 2024; Sudha et al., 2019; AlMalki et al., 2024; Banach and Jedut, 2020; Hadi et al., 2019; Kanazawa et al., 2021; Lauw et al., 2023; Minami et al., 2015; Sato et al., 2024; Tajabadi-Ebrahimi et al., 2017; Wu et al., 2024; Zarrati et al., 2019; Zarrati et al., 2014) were conducted in Asian countries, 6 (Hibberd et al., 2019; Kobyliak et al., 2018; Kopp et al., 2023; Majewska et al., 2020; Michael et al., 2021; Parascinet et al., 2023) in European countries, and 2 (Crovesy et al., 2021; Sergeev et al., 2020) in the United States and Brazil, respectively. The studies were published between 2014 and 2024 and collectively enrolled 1,392 participants. All studies used BMI as the indicator for overweight or obesity. According to the World Health Organization’s classification (WHO, 2024), overweight was defined as a BMI between 25 and 29.9 kg/m^2^, while obesity was confirmed by a BMI of 30 kg/m^2^ or higher. 8 studies (Bai et al., 2024; Sudha et al., 2019; Kanazawa et al., 2021; Minami et al., 2015; Wu et al., 2024; Zarrati et al., 2019; Zarrati et al., 2014; Parascinet et al., 2023) reported the use of *Bifidobacterium breve*, three studies (Hadi et al., 2019; Tajabadi-Ebrahimi et al., 2017; Michael et al., 2021) used *Bifidobacterium longum*, five studies (Sato et al., 2024; Kobyliak et al., 2018; Majewska et al., 2020; Sergeev et al., 2020; AlMalki et al., 2024) used a mixed *Bifidobacterium* species, two studies (Banach and Jedut, 2020; Hibberd et al., 2019) used *Bifidobacterium animalis*, and three studies (Lauw et al., 2023; Kopp et al., 2023; Crovesy et al., 2021) used *Bifidobacterium lactis*. The NIH quality assessment indicated that 11 studies (Bai et al., 2024; AlMalki et al., 2024; Sato et al., 2024; Tajabadi-Ebrahimi et al., 2017; Wu et al., 2024; Zarrati et al., 2019; Zarrati et al., 2014; Kobyliak et al., 2018; Majewska et al., 2020; Michael et al., 2021; Crovesy et al., 2021) had low risk of bias, while 10 studies (Sudha et al., 2019; Banach and Jedut, 2020; Kanazawa et al., 2021; Lauw et al., 2023; Minami et al., 2015; Hibberd et al., 2019; Kopp et al., 2023; Parascinet et al., 2023; Crovesy et al., 2021; Sergeev et al., 2020) exhibited moderate risk of bias, as detailed in Supplementary Table 1.

**Table 2:** Characteristics of studies selected and included in the meta-analysis.

Study	Year	Country	Sample size	Age (Y)	BMI (mean± SD)	Overweight or obese status	Comorbidities	Bifidobacterium strains	Quantity of strains (CFU/day)	Frequency (times/day)	Administration time	Duration (week)	Control Group	Outcomes	NIH
AlMalki et al. (2024)	2024	King Saud	93	19–40	30.8 ± 2.8	Overweight or obese	No	Three bifidobacterium species	30 × 10^9^	2	Week 1	12	Placebo	Weight, BMI, WC, FPG, HbA1c, TC, HDL-C, LDL-C	14
Bai et al. (2024)	2024	China	75	19–45	31.4 ± 3.1	Overweight or obese	No	*Bifidobacterium breve*	10^10^	1	Week 1	12	Placebo	Weight, BMI, FPG, TC, TG, HDL-C, LDL-C	12
Banach and Jedut (2020)	2020	China	54	20–49	34.9 ± 3.9	Obese	No	*Bifidobacterium animalis* subsp	NI	1	Week 1	12	The same diet group without probiotics	Weight, BMI	9
Crovesy et al. (2021)	2021	Brazil	32	19–40	35.0 ± 7.5	Obese	No	*Bifidobacterium lactis*	10^9^	1	Week 1	8	No specific treatment	Weight, BMI, WC	6
Hadi et al. (2019)	2019	Iran	60	20–65	30.9 ± 3.5	Overweight or obese	No	*Bifidobacterium bifidum*	2 × 10^9^	1	Week 1	8	Placebo	Weight, BMI, WC, FPG, TC, TG, LDL-C, HDL-C,	11
Hibberd et al. (2019)	2019	Finland	134	20–49	31.1 ± 1.9	Overweight or obese	No	*Bifidobacterium animalis* subsp.	10^10^	1	Week 1	24	Placebo	Weight, BMI, WC, HbA1c, Insulin, TC, TG, HDL-C, LDL-C	8
Kanazawa et al. (2021)	2021	Japan	88	30–80	29.3 ± 4.1	Overweight or obese	T2DM	*Bifidobacterium breve*	3×10^8^	2	Week 1	24	Control group without probiotics	BMI, FPG, HbA1c, TC, TG, HDL-C	6
Kobyliak et al. (2018)	2018	Ukraine	53	18–75	35.1 ± 1.48	Obese	T2DM	Bifidobacterium	10^10^	1	Week 1	8	Placebo	Weight, BMI, WC, FPG, HbA1c, Insulin	14
Kopp et al. (2023)	2023	Germany	76	25–65	34.3 ± 3.3	Obese	No	bifidobacterium lactis	1.25 × 10^9^	1	Week 1	8	Placebo	Weight, BMI, InsulinP	9
Lauw et al. (2023)	2023	China	55	20–65	29.3 ± 4.0	Overweight or obese	No	*Bifidobacterium lactis*	5 × 10^9^	2	Week 1	8	Dietary intervention group	Weight, BMI, Insulin, TC, TG, HDL-C	7
Majewska et al. (2020)	2020	Poland	50	45–70	36.4 ± 5.2	Obese	No	*Bifidobacterium bifidum*, *Bifidobacterium lactis*	2.5 × 10 ^9^	1	Week 1	12	Placebo	TC, TG, HDL-C, LDL-C	11
Michael et al. (2021)	2021	Bulgaria	70	45–65	28 ± 1.5	Overweigh	No	Bifdobacterium bifdum	5 × 10^10^	1	Week 1	12	Placebo	Weight, BMI, WC	13
Minami et al. (2015)	2015	Japan	52	40–69	27.4 ± 0.6	Overweigh	No	*Bifidobacterium breve*	5 × 10^10^	1	Week 1	12	Placebo	Weight, BMI, FPG, HbA1c, Insulin	9
Parascinet et al. (2023)	2023	Spain	20	≥18	≥30	obese	No	*Bifidobacterium breve*	10^9^	1	Week 1	10	Control group	FPG, HbA1c, TC, TG, HDL-C, LDL-C	9
Sato et al. (2024)	2024	Japan	100	20–64	26.1 ± 1.8	Overweight	No	*Bifidobacterium longum*, *Bifidobacterium breve*	1 × 10^10^ and 5 × 10^10^	2	Week 1	16	Placebo	Weight, BMI, TG	12
Sergeev et al. (2020)	2020	America	20	30–65	33.5 ± 5.0	Obese	No	*Bifidobacterium lactis*, *Bifidobacterium longum*, *Bifidobacterium bifidum*	15 × 10^10^	1	Week 1	12	Placebo	Weight, BMI, WC, HbA1c	9
Sudha et al. (2019)	2019	India	71	30–65	27.6 ± 2.2	Overweight	No	*Bifidobacterium breve*	5 × 10^9^	1	Week 1	12	Placebo	Weight, BMI, FPG, TC, TG, HDL-C	9
Tajabadi-Ebrahimi et al. (2017)	2016	King Saud	60	40–85	31.0 ± 5.5	Overweight or obese	T2DM	*Bifidobacterium bifidum*	2 × 10^9^	1	Week 1	12	Placebo	Weight, BMI, FPG, Insulin, TC, TG, HDL-C, LDL-C	11
Wu et al. (2024)	2024	China	75	19–45	31.4 ± 3.1	Overweight or obese	No	*Bifidobacterium breve*	10^10^	1	Week 1	12	Placebo	Weight, BMI, FPG, TC, TG, HDL-C, LDL-C	11
Zarrati et al. (2019)	2018	Iran	60	20–50	32.1 ± 4.4	Overweight or obese	No	*Bifidobacterium bifidum*	10 ^8^	2	Week 1	8	Conventional yogurt group	Weight, BMI, WC	11
Zarrati et al. (2014)	2014	Iran	75	20–50	33.9 ± 6.5	Overweight or obese	No	*Bifidobacterium bifidum*	10 ^7^	1	Week 1	8	Regular yogurt group	Weight, BMI, WC	12

BMI, body mass index; WC, waist circumference; FBG, fasting blood glucose; HbA1c, glycated hemoglobin; TC, total cholesterol; TG, triglyceride; HDL-C, high density lipoprotein cholesterol; LDL-C, low density lipoprotein cholesterol.

### 3.3 Primary outcomes

#### 3.3.1 Effects of *Bifidobacterium* on weight management

**Figure 2:**
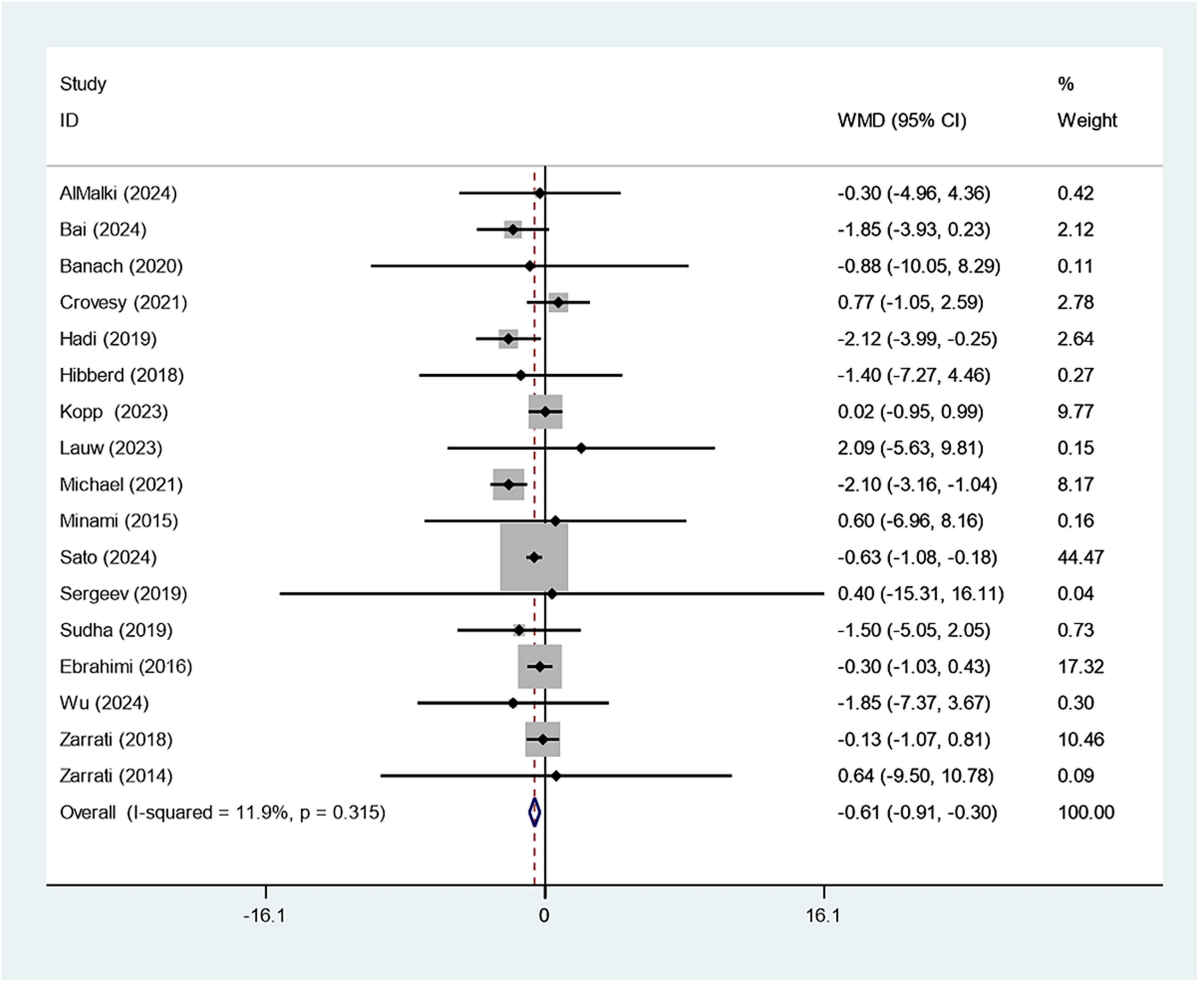
Forest plot for weight.

Eighteen studies reported on the relationship between *Bifidobacterium* supplementation and weight change. Sensitivity analysis demonstrated that the study conducted by Kobyliak had a considerable impact on the overall findings, as illustrated in Supplementary Figure 1. Upon exclusion of this study, the experimental group receiving *Bifidobacterium* supplementation showed a significantly greater reduction in body weight compared to the control group (WMD: −0.607 kg; 95% CI: −0.910,–0.303; *I^2^* = 11.9%), as shown in Figure 2. Funnel plot symmetry and the Egger test (*p* = 0.804) suggested no significant publication bias, as presented in Supplementary Figure 2.

**Figure 3:**
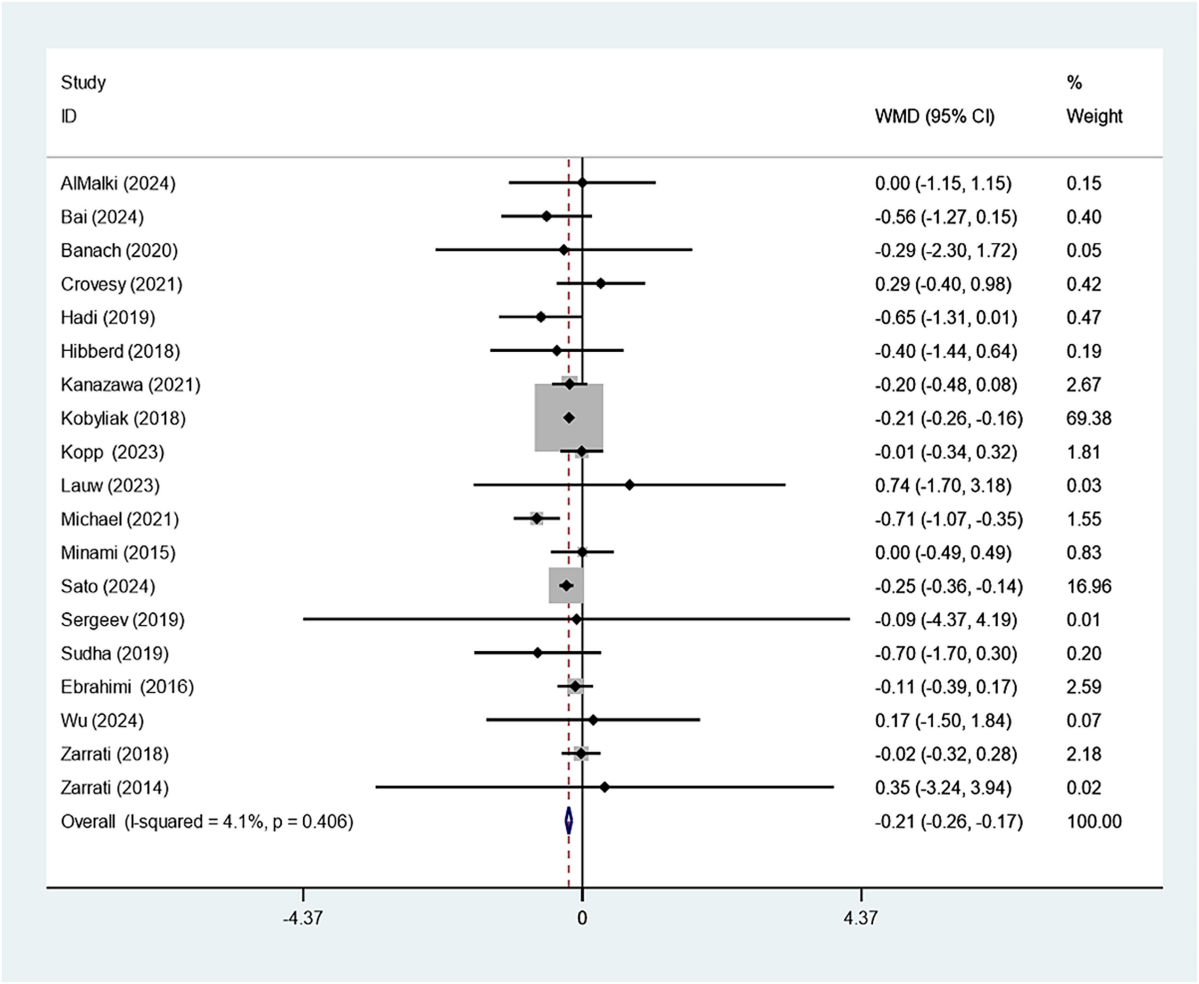
Forest plot for BMI.

**Figure 4:**
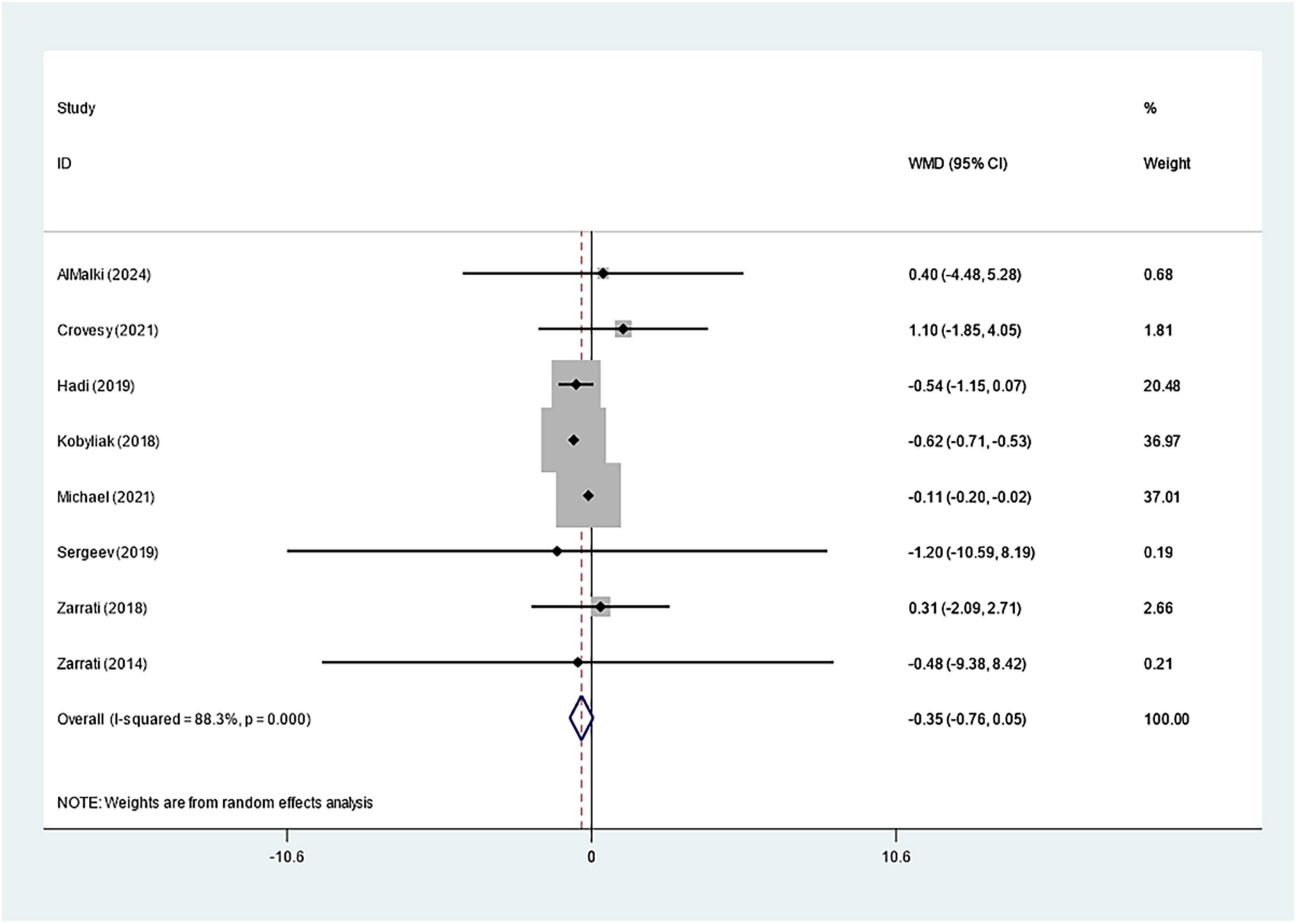
Forest plot for waist circumference.

19 studies explored how *Bifidobacterium* supplementation influences BMI. Our meta-analysis demonstrated a notably larger BMI decline in the experimental group relative to the control (WMD: −0.214 kg/m^2^; 95% CI: −0.259, −0.169, *I^2^* = 4.1%) (Figure 3). Eight studies examined the impact of *Bifidobacterium* on WC, but the difference across groups was insignificant (WMD: −0.353 cm; 95% CI: −0.759, −0.053, *I^2^* = 88.3%) (Figure 4). Sensitivity analyses for BMI and WC indicated robust results, as shown in Supplementary Figures 3, 4. Both the funnel plot and Egger’s test (*p* = 0.867) demonstrated no discernible publication bias (Supplementary Figure 5).

### 3.4 Secondary outcomes

#### 3.4.1 Effects of *Bifidobacterium* on glycemic control

**Figure 5:**
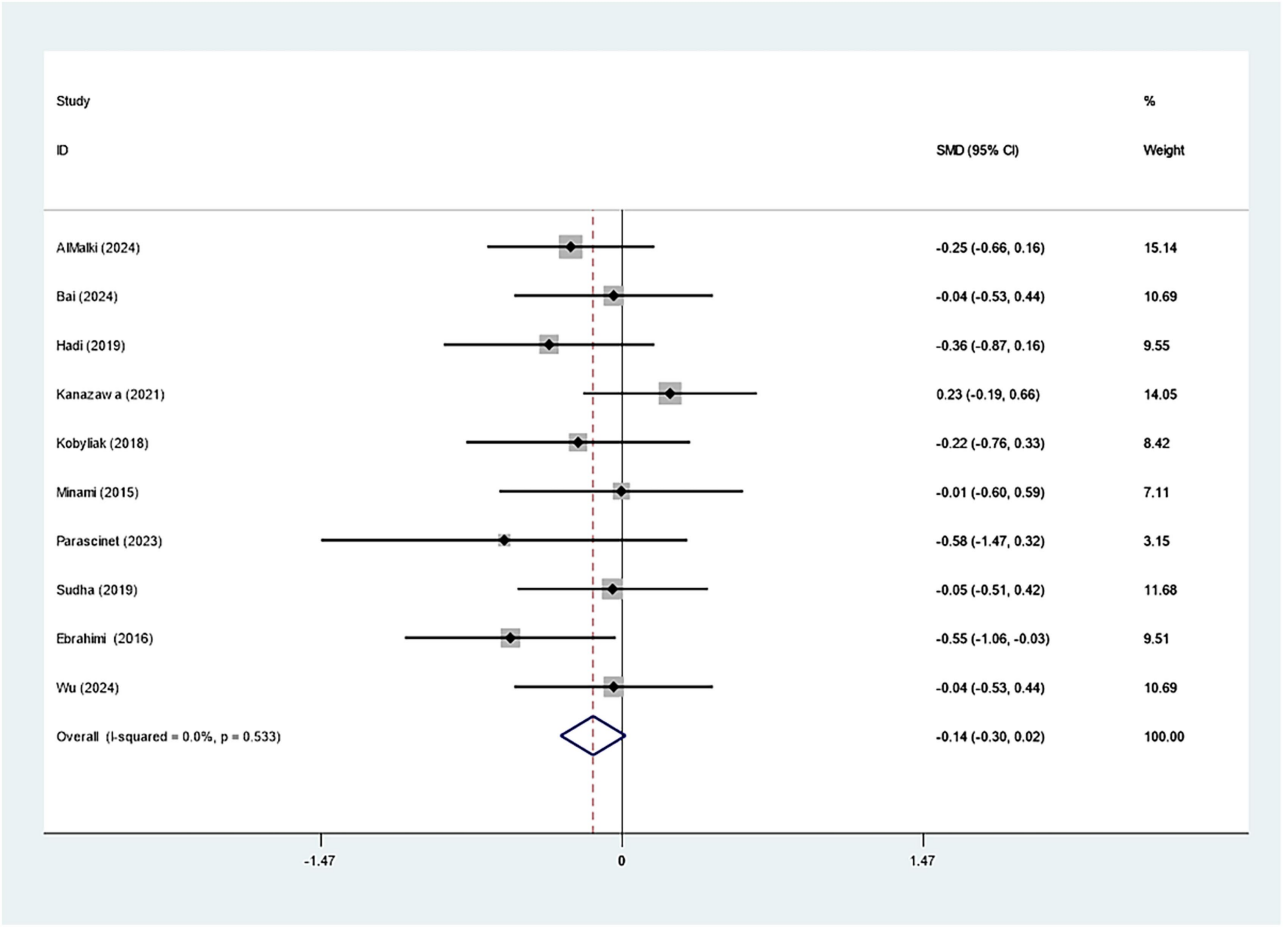
Forest plot for fasting blood glucose.

Ten studies evaluated how *Bifidobacterium* supplementation influences FBG within the cohort with excess weight or obesity. *Bifidobacterium* did not markedly affect FBG (SMD: -0.143; 95% CI: −0.302, −0.016, *I^2^* = 0.0%) (Figure 5). Our sensitivity analysis indicated robustness. Egger’s test (*p* = 0.215) and the funnel plot demonstrated no publication bias, as depicted in Supplementary Figures 6, 7.

**Figure 6:**
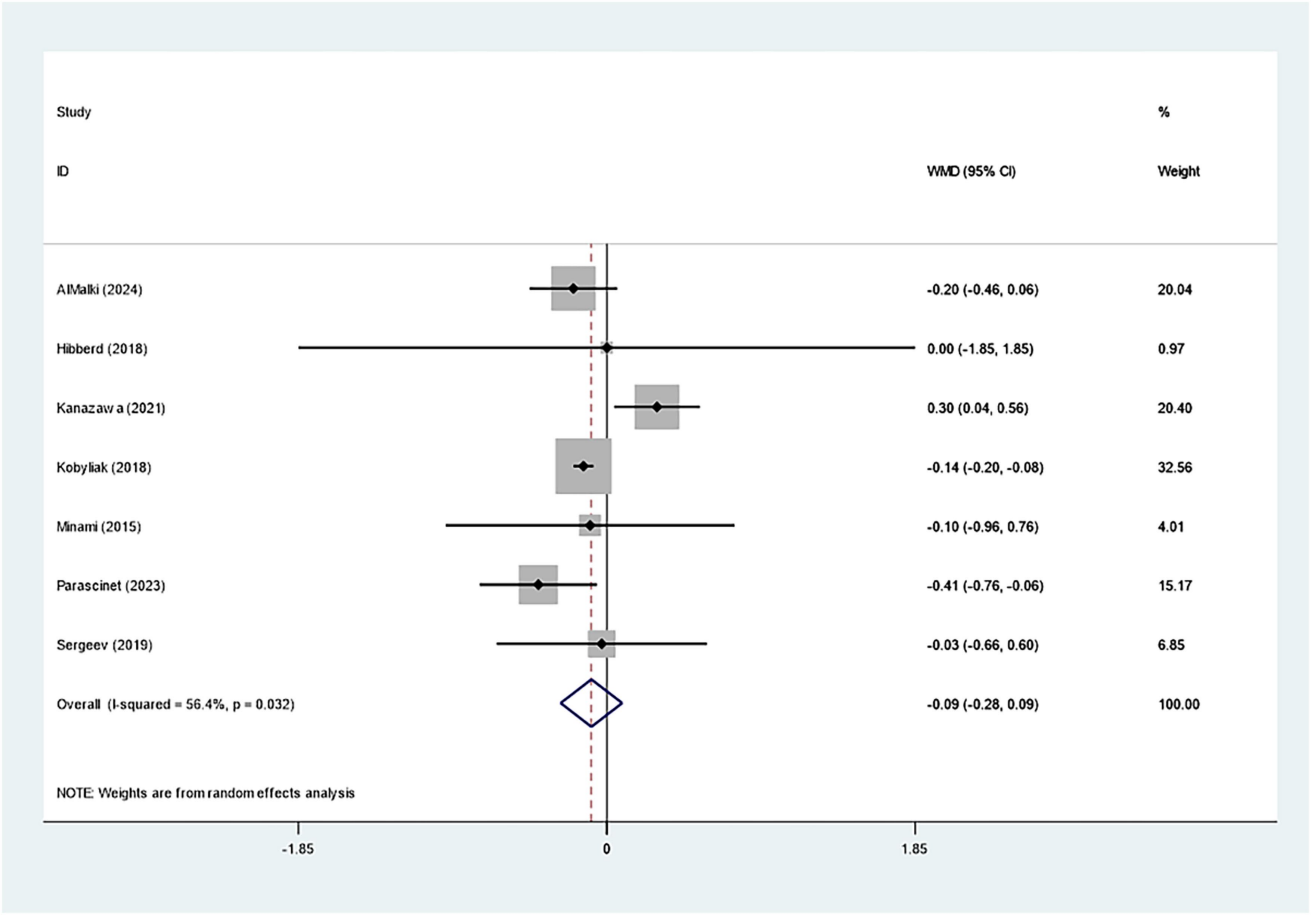
Forest plot for glycated hemoglobin.

**Figure 7:**
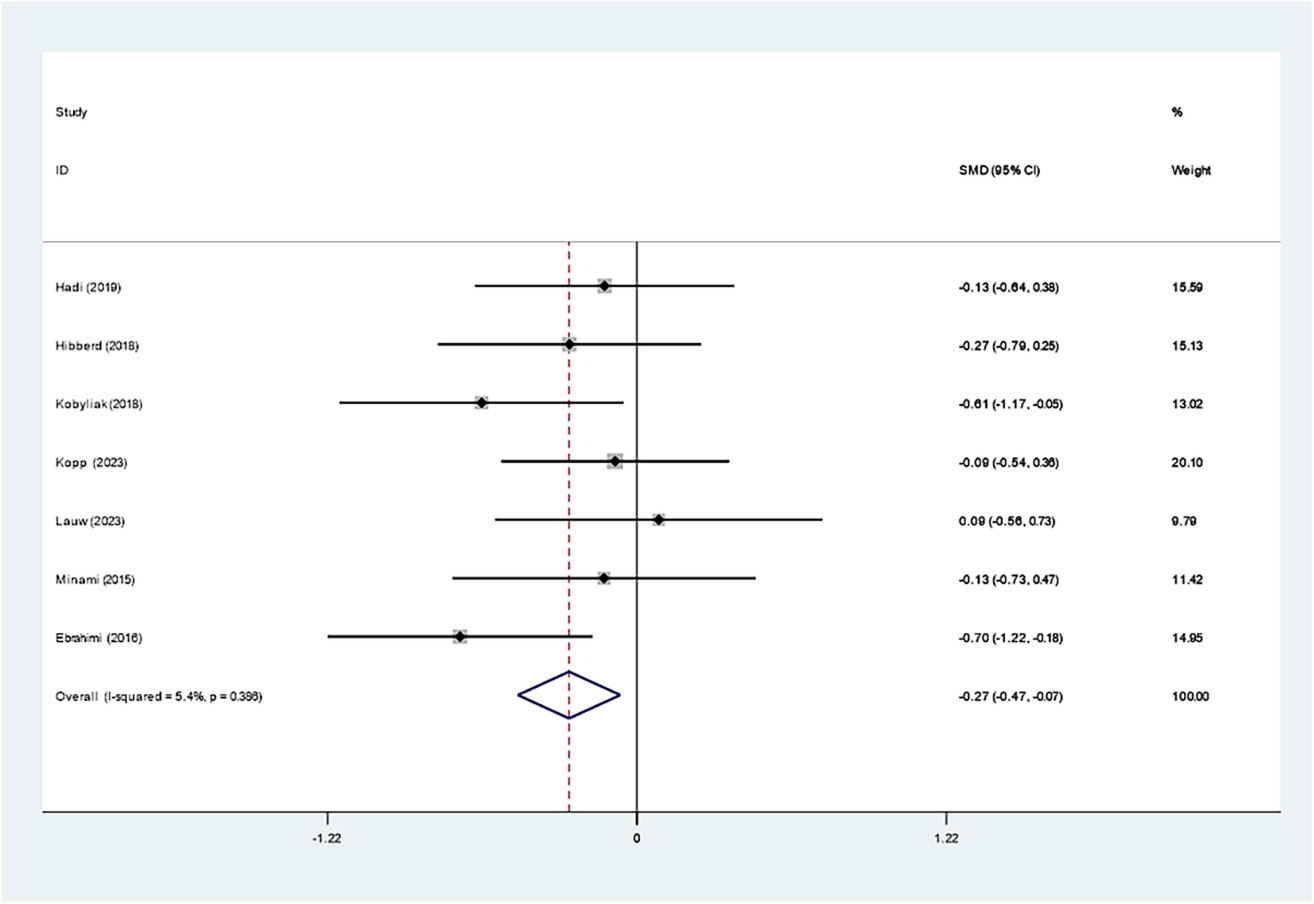
Forest plot for insulin.

Seven studies investigated the effect of *Bifidobacterium* on HbA1c, demonstrating insignificant variations across groups (WMD: −0.093%; 95% CI: −0.277, −0.091, *I^2^* = 56.4%) (Figure 6). However, regarding insulin levels, seven studies reported a notable decrease in insulin levels in the *Bifidobacterium* supplementation cohort (SMD: −0.268; 95% CI: −0.470, −0.066, *I^2^* = 5.4%) (Figure 7). These findings were robust after sensitivity analysis, as presented in Supplementary Figures 8, 9.

#### 3.4.2 Effects of *Bifidobacterium* on lipid metabolism

To unravel how *Bifidobacterium* impacts lipid metabolism, our study incorporated studies evaluating TC, TG, HDL-C, and LDL-C. The effects on lipid levels were insignificant.

**Figure 8:**
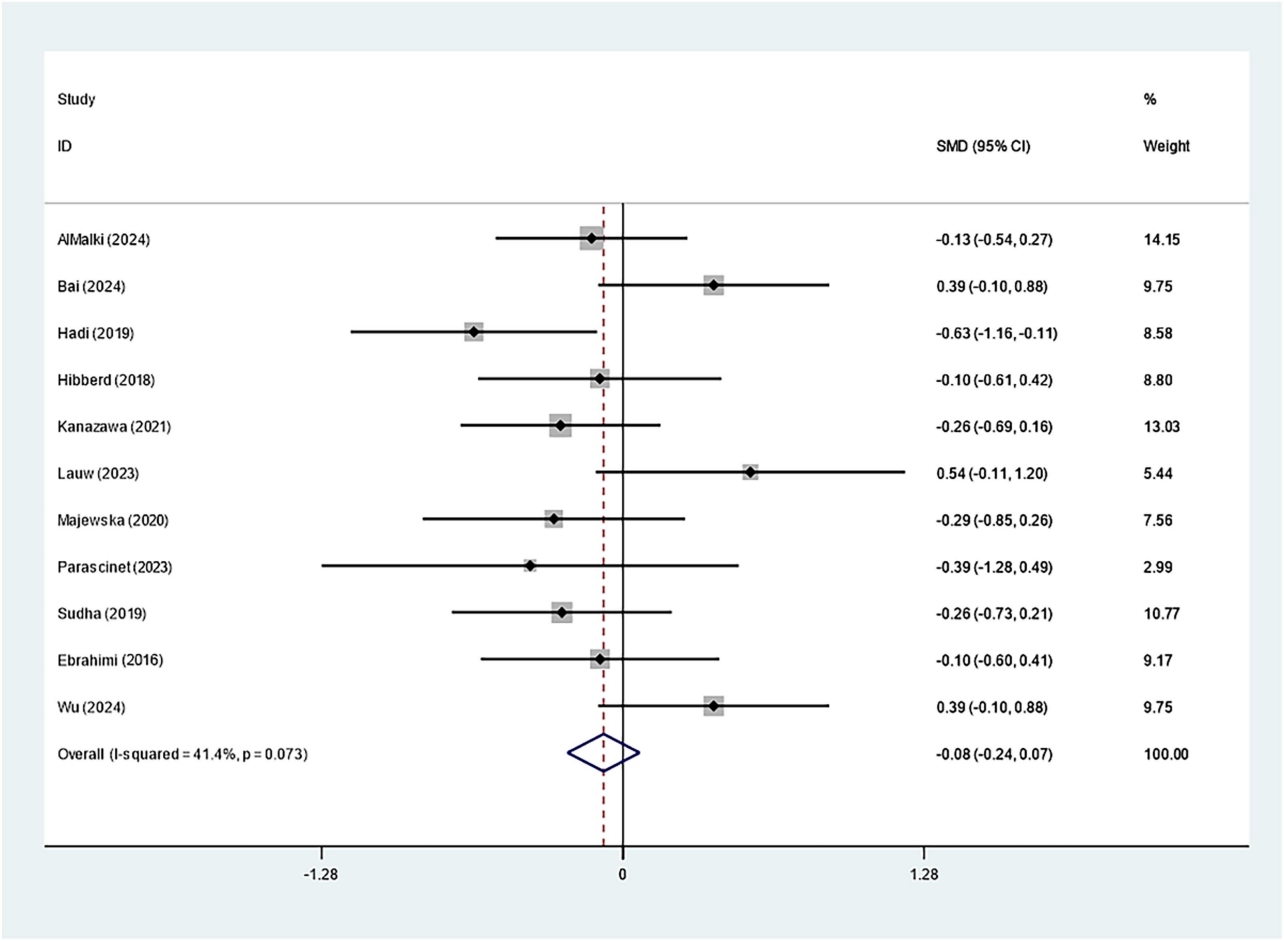
Forest plot for total cholesterol.

**Figure 9:**
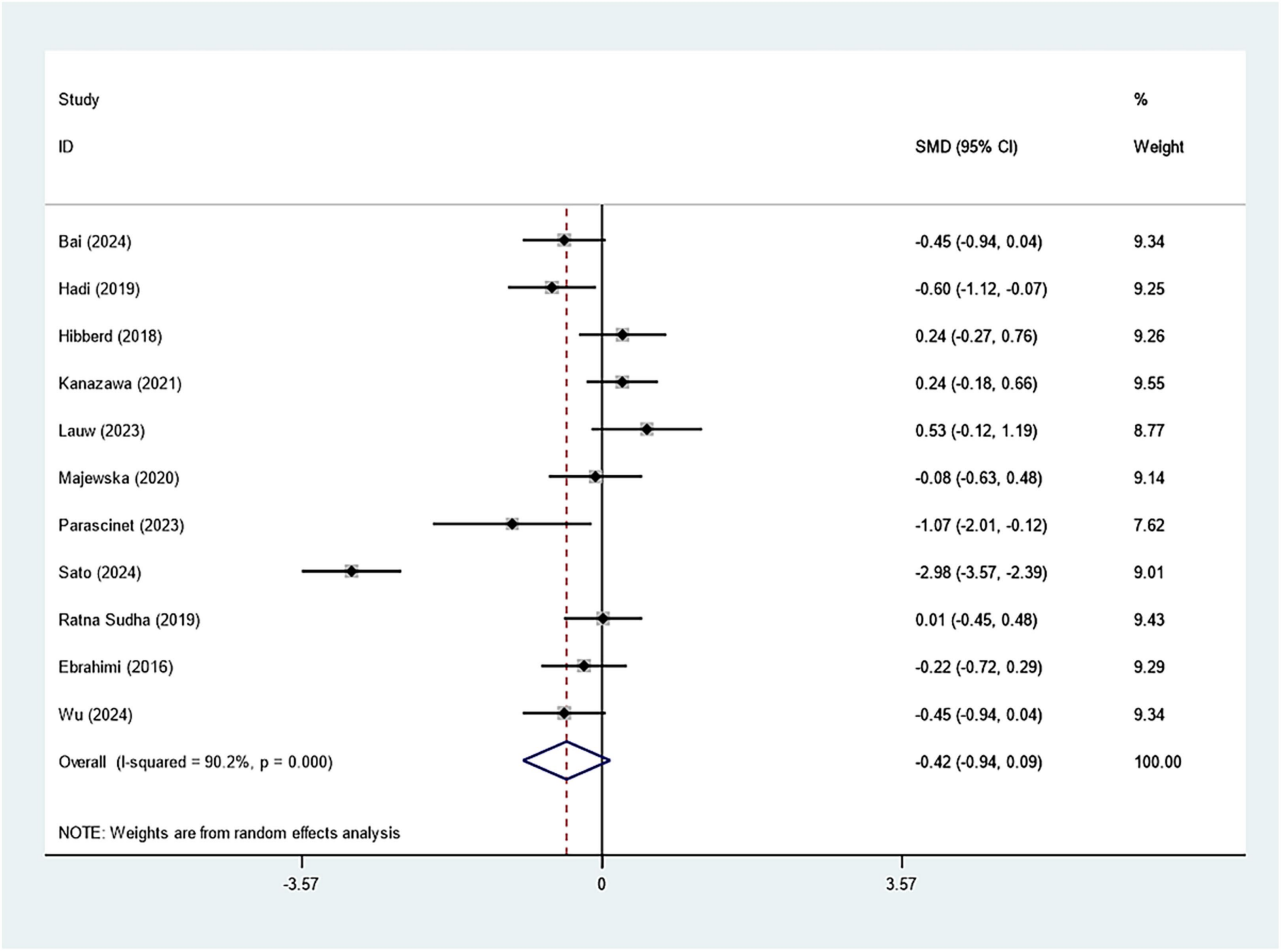
Forest plot for triglycerides.

**Figure 10:**
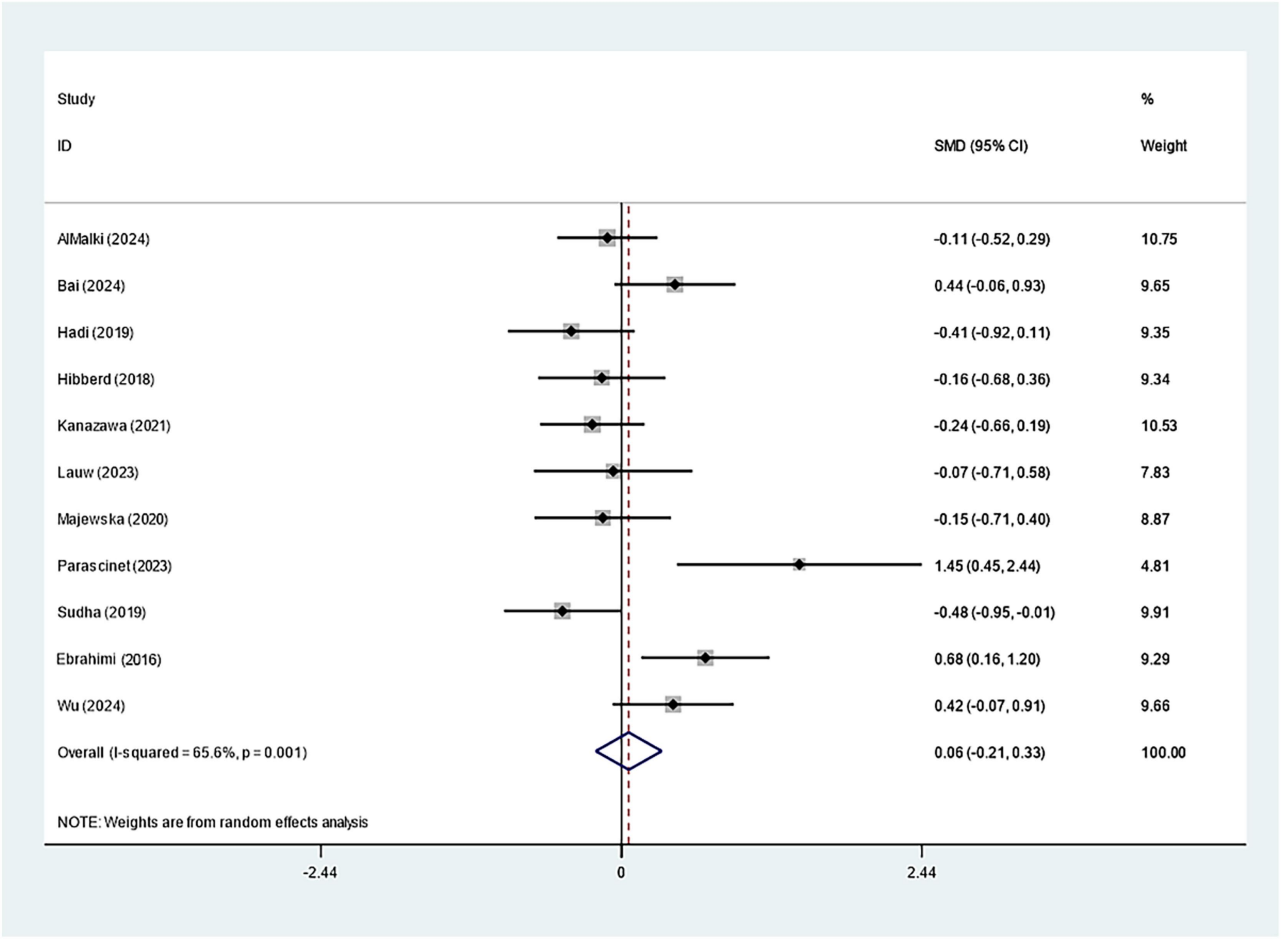
Forest plot for high-density lipoprotein.

Specifically, eleven studies regarding TC, TG, and HDL-C suggested insignificant effects of *Bifidobacterium* supplementation (TC: SMD: -0.082; 95% CI: −0.235, 0.071, *I^2^* = 41.4%; TG: SMD: -0.423; 95% CI: −0.939, 0.094, *I^2^* = 90.2%; HDL-C: SMD: 0.060; 95% CI: −0.209, 0.328, *I^2^* = 65.6%) (Figures 8–10). The robustness of the foregoing results was proved by utilizing sensitivity analysis. Publication bias did not exist in funnel plots and Egger’s test (Supplementary Figures 10–15).

**Figure 11:**
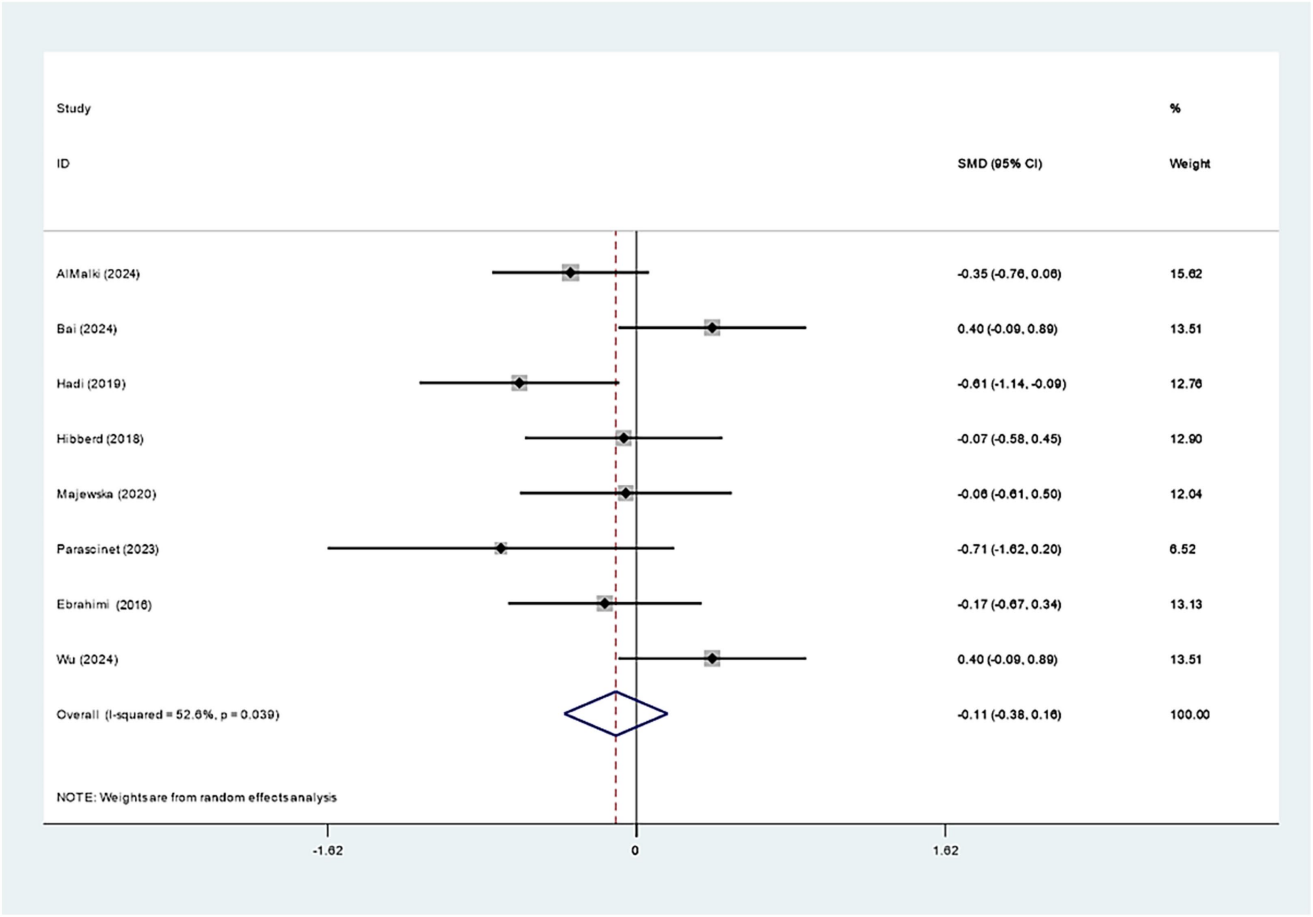
Forest plot for low-density lipoprotein.

Eight studies examined the impact of *Bifidobacterium* on LDL-C and revealed no evident effect (SMD: -0.108; 95% CI: −0.379, 0.162, *I^2^* = 52.6%) (Figure 11). Sequential exclusion of individual studies in sensitivity analyses did not markedly affect the results, as presented in Supplementary Figure 16.

## 4 Discussion

This meta-analysis assessed the impact of *Bifidobacterium* supplementation on individuals with overweight or obesity. Our study on 21 RCTs proved that *Bifidobacterium* supplementation is effective in managing weight of the overweight or obese patients. The *Bifidobacterium* cohort had a prominent decline in weight and BMI, though no significant effect on WC was observed. Furthermore, supplementation with *Bifidobacterium* did not notably lower FBG or HbA1c but was linked to improved insulin levels. In contrast, marked differences were not found across the two groups with regard to TC, TG, HDL-C, and LDL-C. Therefore, *Bifidobacterium* supplementation is beneficial for overweight or obese individuals, potentially aiding in weight control and improving insulin secretion.

Probiotics, including *Bifidobacterium*, have been shown to help manage overweight and obesity (Geng et al., 2022). Our findings prove that *Bifidobacterium* supplementation lowers the weight and BMI of the population with excess weight or obesity, which aligns with previous research results (Sadeghi et al., 2024). *Bifidobacterium* may promote the production of SCFAs, such as acetate, propionate, and butyrate, in the gut. These SCFAs can activate the AMPK pathway in the liver, muscle, and adipose tissues. AMPK phosphorylates acetyl-CoA carboxylase (ACC), thereby inhibiting fatty acid synthesis, while simultaneously activating carnitine palmitoyltransferase-1 (CPT-1), which facilitates the mitochondrial uptake and oxidation of fatty acids, enhancing lipid catabolism (Yun et al., 2024). In overweight or obese individuals, sustained supplementation with *Bifidobacterium* may reduce excessive lipid absorption. Furthermore, SCFAs can activate free fatty acid receptors (FFAR2/3) on intestinal epithelial cells, stimulating the secretion of glucagon-like peptide-1 (GLP-1) and peptide YY (PYY), which suppress the release of the appetite-stimulating hormone ghrelin (Horiuchi et al., 2020). This leads to reduced food intake and subsequent caloric restriction, thereby contributing to a natural decline in BMI. Individuals with obesity often exhibit lower levels of SCFAs produced by gut microbiota; supplementation with probiotics such as *Bifidobacterium* can enhance SCFA production, increase satiety, and promote weight reduction. López-Moreno et al. (2020) noted marked weight and BMI drops in a cohort receiving probiotics containing *Bifidobacterium* in contrast to a placebo cohort. Nevertheless, this meta-analysis found no significant reduction in WC among overweight or obese individuals receiving *Bifidobacterium* supplementation. Ejtahed et al. (2019) demonstrated that probiotic intervention over 2–24 weeks was effective in reducing WC in overweight or obese subjects, which may be attributed to the relatively shorter intervention period (less than 12 weeks) in the present study. Additionally, there exist gender differences in probiotic effects on weight management among the overweight or obese population, with some studies noting a reduction in WC only in women (Cao et al., 2024). This may also explain the insignificant effect on WC in our findings.

Our study demonstrated that supplementation with *Bifidobacterium* significantly reduced insulin levels in overweight or obese individuals. This effect may be attributed to the capacity of *Bifidobacterium* to reshape the composition and function of the gut microbiota. In such populations, its metabolic byproducts, particularly SCFAs, may activate the gut-metabolism axis, enhance insulin signaling, reduce adipose tissue accumulation, improve insulin sensitivity, and ultimately lower the risk of obesity-related diseases (Guney-Coskun and Basaranoglu, 2024). Teo et al. (2024) also proved that probiotics containing *Bifidobacterium* were more effective in improving insulin levels. Although the present study supports the beneficial role of *Bifidobacterium* in modulating insulin levels, the differences in the results are relatively small. Further randomized controlled trials and prospective cohort studies are warranted to elucidate the role of Bifidobacterium in mitigating insulin resistance among obese populations. Furthermore, this meta-analysis found no significant changes in FBG or HbA1c levels. Barengolts et al. (2019) also reported no changes in HbA1c, FBG, or fasting insulin following consumption of *Bifidobacterium*-containing probiotic yogurt in the obese cohort with T2DM in comparison to traditional yogurt. This may be because *Bifidobacterium*’s effect on postprandial blood glucose is more prominent. Therefore, additional research is necessitated to verify the efficacy of *Bifidobacterium* supplementation in managing blood glucose levels in overweight or obese individuals.

This study found no significant effect of *Bifidobacterium* on lipid metabolism in the experimental group, suggesting that *Bifidobacterium* may primarily exert its effects through energy metabolism rather than lipid redistribution. Dong et al.’s meta-analysis (Dong et al., 2019) also reported insignificant disparities in TC, TG, or HDL between the intervention group (which used probiotic foods and *Bifidobacterium*-containing supplements) and the control group in individuals with metabolic syndrome. Additionally, other studies (Ruscica et al., 2019) have indicated that *Bifidobacterium longum* evidently reduces serum TC and LDL-C in the hyperlipidemic population by influencing gut microbiota composition and fecal metabolic products. This discrepancy possibly arises from differences in the specific strains of *Bifidobacterium* in this study, as the lipid-lowering effects of different *Bifidobacterium* strains may vary. Although our study showed no significant effect on these lipid parameters, interest in the role of *Bifidobacterium* in lipid metabolism remains strong in the academic community. High-quality RCTs are necessitated to better elucidate the efficacy of *Bifidobacterium* in lipid metabolism.

This study has two limitations. First, heterogeneity in the studies on *Bifidobacterium* may confound the results due to differences in ethnicity, sex, strain types, and dosages. Second, the lack of standardization in the units of measurement for blood glucose and lipids may introduce measurement errors. However, efforts were made in this study to minimize such effects through standardization.

## 5 Conclusion

In conclusion, this study suggests that supplementation with *Bifidobacterium* exerts a moderate positive effect on lowering the weight and BMI of overweight or obese people, indicating that *Bifidobacterium* may serve as an adjunct in weight management for these individuals. Additionally, our findings demonstrate a beneficial role of *Bifidobacterium* in regulating insulin levels in overweight or obese patients, though its effects on improving hyperglycemia and hyperlipidemia were limited. Future studies should focus on high-quality clinical trials that consider individual factors such as gender, fat distribution, and strain specificity. Specific *Bifidobacterium* strains may have superior effects, and more research is necessitated to verify the role of *Bifidobacterium* in managing weight, providing valuable insights into its potential applications.

## Data Availability

The original contributions presented in the study are included in the article/Supplementary material, further inquiries can be directed to the corresponding author.
