# Supplementary material for: Effects of Bifidobacterium on metabolic parameters in overweight or obesity adults: a systematic review and meta-analysis
Source: Front Microbiol. 2025 Sep 25;16:1633434. doi: 10.3389/fmicb.2025.1633434 (PMC12509062; doi:10.3389/fmicb.2025.1633434)
Supplement: Supplementary file 1 [file Supplementary_file_1.docx]

**Supplementary materials**

**Supplementary table 1** NIH Systematic Review Quality Assessment Form

| ID | Q1 | Q2 | Q3 | Q4 | Q5 | Q6 | Q7 | Q8 | Q9 | Q10 | Q11 | Q12 | Q13 | Q14 | Total |
| --- | --- | --- | --- | --- | --- | --- | --- | --- | --- | --- | --- | --- | --- | --- | --- |
| AlMalk (2024) | 1 | 1 | 1 | 1 | 1 | 1 | 1 | 1 | 1 | 1 | 1 | 1 | 1 | 1 | 14 |
| Bai (2024) | 1 | 1 | 1 | 1 | 1 | 1 | 1 | 1 | 0 | 1 | 1 | 1 | 1 | 0 | 12 |
| Banach (2020) | 1 | 1 | 0 | 0 | 0 | 1 | 1 | 1 | 0 | 1 | 1 | 1 | 1 | 0 | 9 |
| Crovesy (2021) | 1 | 0 | 0 | 0 | 0 | 0 | 1 | 1 | 0 | 1 | 1 | 0 | 1 | 0 | 6 |
| Hadi (2019) | 1 | 1 | 1 | 1 | 0 | 1 | 1 | 1 | 0 | 1 | 1 | 1 | 1 | 0 | 11 |
| Hibberd (2018) | 1 | 0 | 0 | 1 | 0 | 1 | 1 | 1 | 0 | 1 | 1 | 0 | 1 | 0 | 8 |
| Kanazawa (2021) | 1 | 0 | 0 | 0 | 0 | 0 | 1 | 1 | 0 | 1 | 1 | 0 | 1 | 0 | 6 |
| Kobyliak (2018) | 1 | 1 | 1 | 1 | 1 | 1 | 1 | 1 | 1 | 1 | 1 | 1 | 1 | 1 | 14 |
| Kopp (2023) | 1 | 0 | 0 | 1 | 0 | 1 | 1 | 1 | 0 | 1 | 1 | 1 | 1 | 0 | 9 |
| Lauw (2023) | 1 | 1 | 1 | 0 | 0 | 1 | 0 | 0 | 0 | 1 | 1 | 0 | 1 | 0 | 7 |
| Majewska (2020) | 1 | 0 | 0 | 1 | 0 | 1 | 1 | 1 | 1 | 1 | 1 | 1 | 1 | 1 | 11 |
| Michael (2021) | 1 | 1 | 1 | 1 | 1 | 0 | 1 | 1 | 1 | 1 | 1 | 1 | 1 | 1 | 13 |
| Minami (2015) | 1 | 0 | 0 | 1 | 0 | 1 | 1 | 1 | 1 | 1 | 1 | 0 | 1 | 0 | 9 |
| Parascinet (2023) | 1 | 0 | 0 | 0 | 0 | 1 | 1 | 1 | 1 | 1 | 1 | 0 | 1 | 1 | 9 |
| Sato (2024) | 1 | 1 | 1 | 1 | 1 | 1 | 1 | 1 | 0 | 1 | 1 | 1 | 1 | 0 | 12 |
| Sergeev (2020) | 1 | 0 | 0 | 0 | 0 | 1 | 1 | 1 | 1 | 1 | 1 | 0 | 1 | 1 | 9 |
| Sudha (2019) | 1 | 1 | 1 | 1 | 0 | 1 | 0 | 0 | 1 | 1 | 1 | 0 | 1 | 0 | 9 |
| Ebrahimi (2016) | 1 | 1 | 1 | 1 | 0 | 1 | 1 | 1 | 0 | 1 | 1 | 1 | 1 | 0 | 11 |
| Wu (2024) | 1 | 1 | 1 | 1 | 0 | 1 | 1 | 1 | 0 | 1 | 1 | 0 | 1 | 1 | 11 |
| Zarrati (2018) | 1 | 1 | 1 | 1 | 0 | 1 | 1 | 1 | 0 | 1 | 1 | 0 | 1 | 1 | 11 |
| Zarrati (2014) | 1 | 1 | 1 | 1 | 0 | 1 | 1 | 1 | 1 | 1 | 1 | 0 | 1 | 1 | 12 |

**Supplementary Figures**


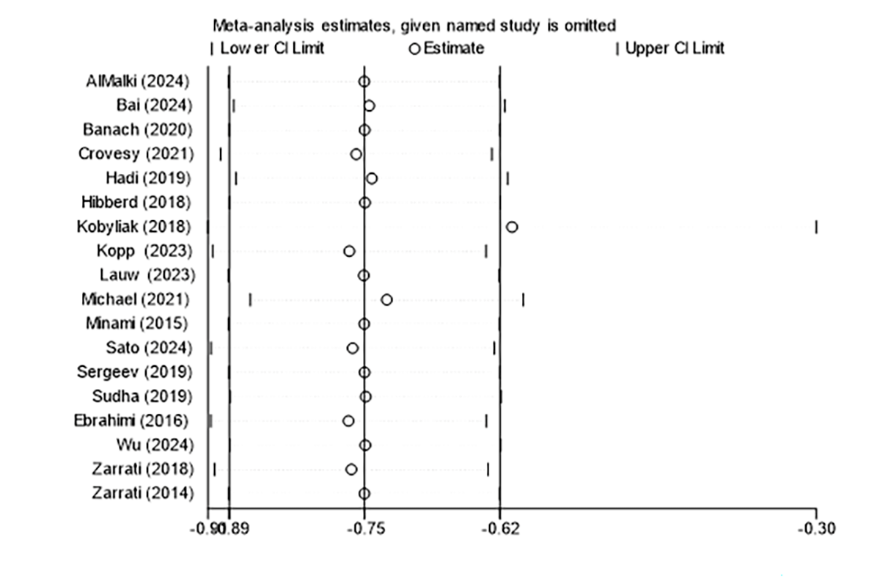


**Supplementary Figure 1** Sensitivity analysis of body weight


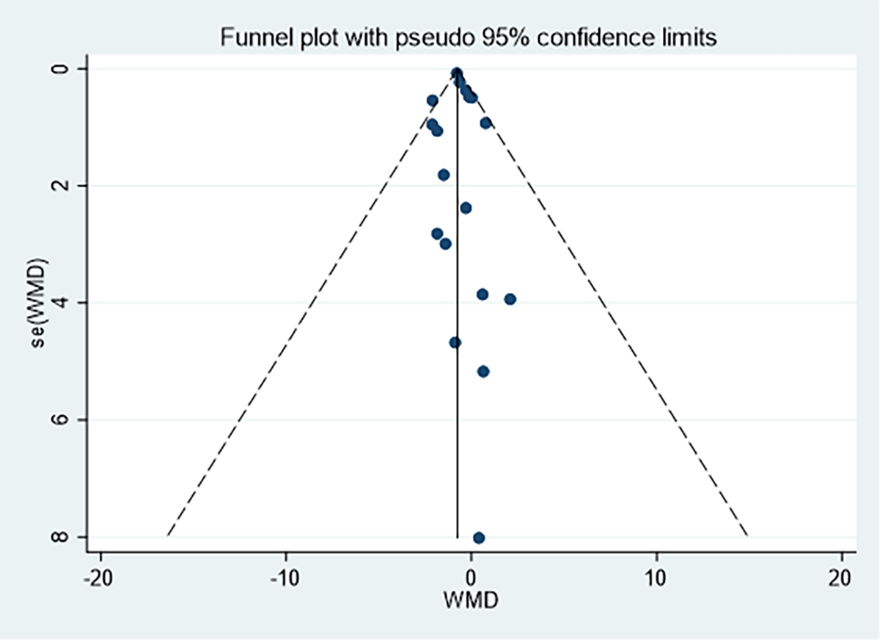


**Supplementary Figure 2** Funnel plot of weight


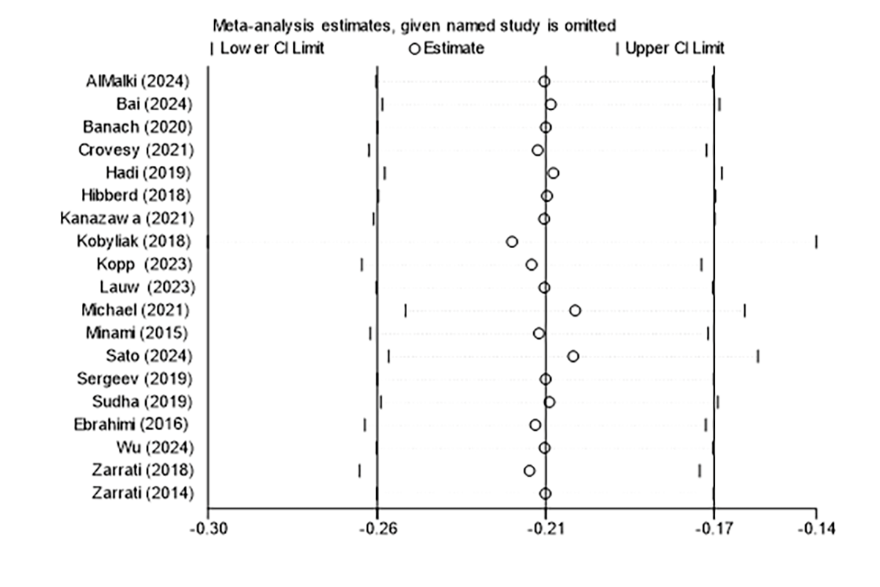


**Supplementary Figure 3** Sensitivity analysis of BMI


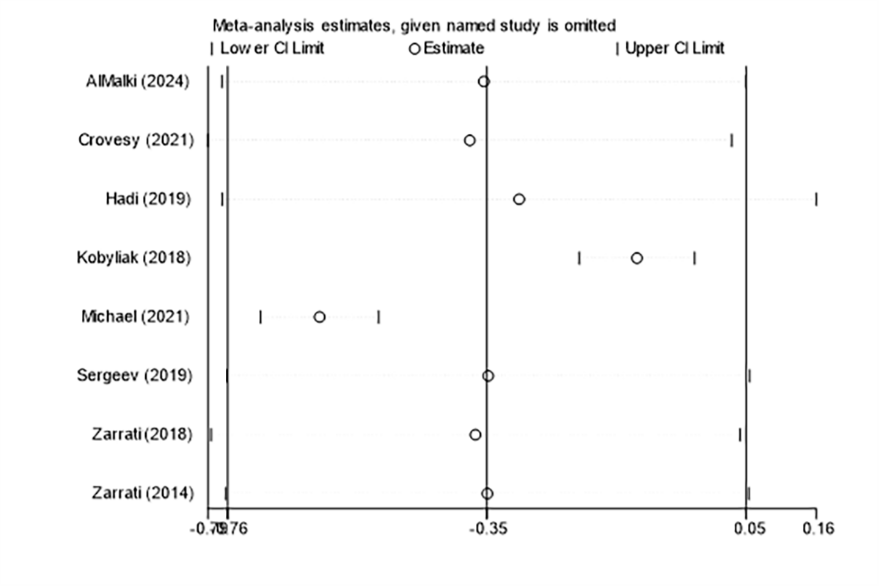


**Supplementary Figure 4** Sensitivity analysis of waist Circumference


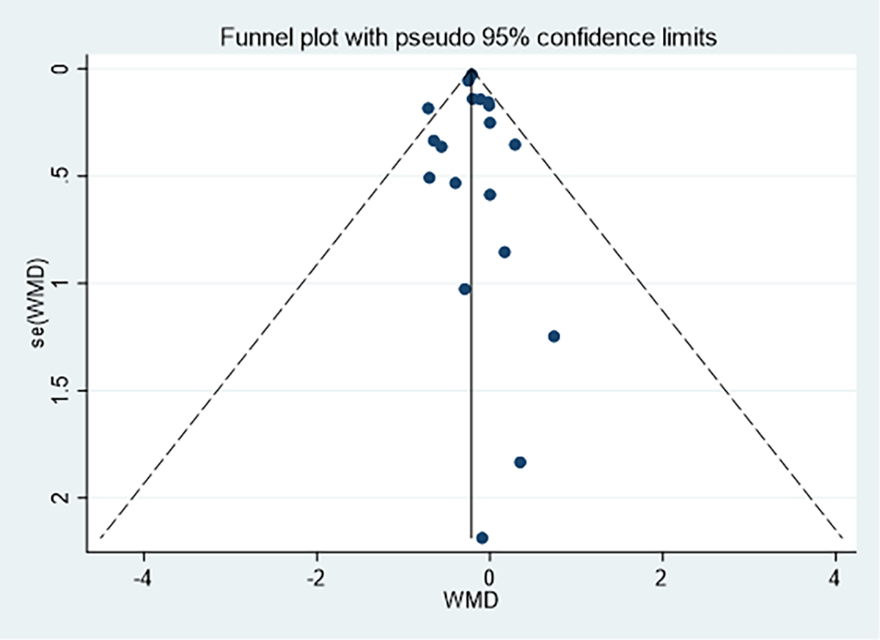


**Supplementary Figure 5** The funnel plot of BMI


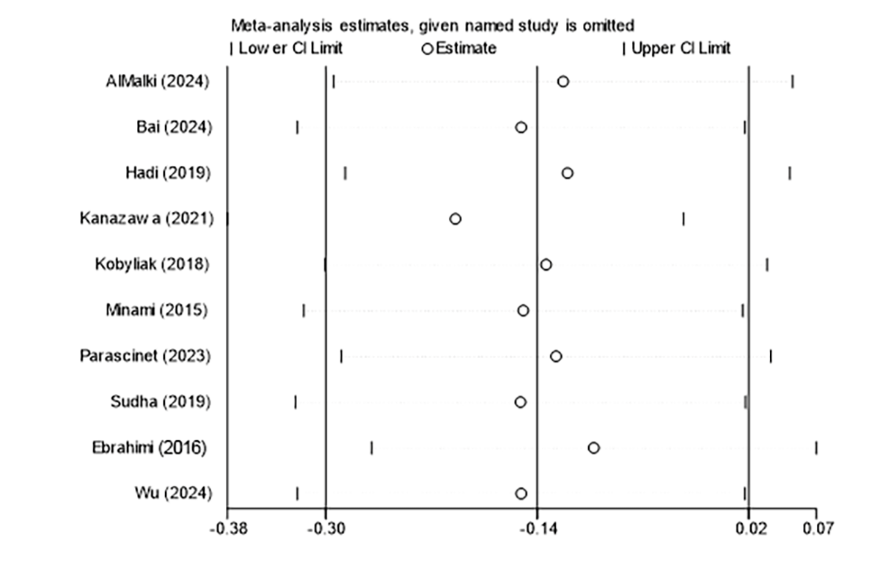


**Supplementary Figure 6** Sensitivity analysis of fasting blood glucose


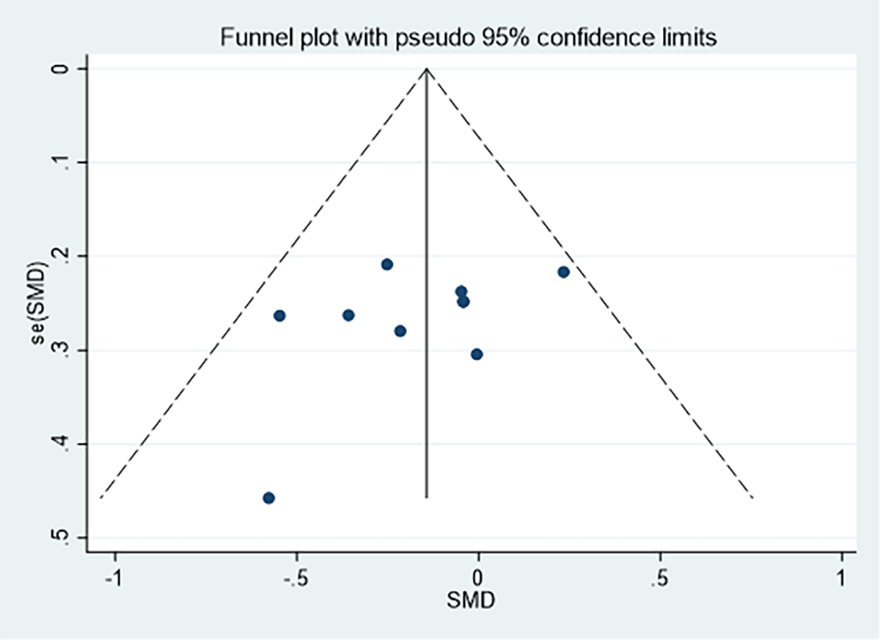


**Supplementary Figure 7** Funnel plot of fasting blood glucose


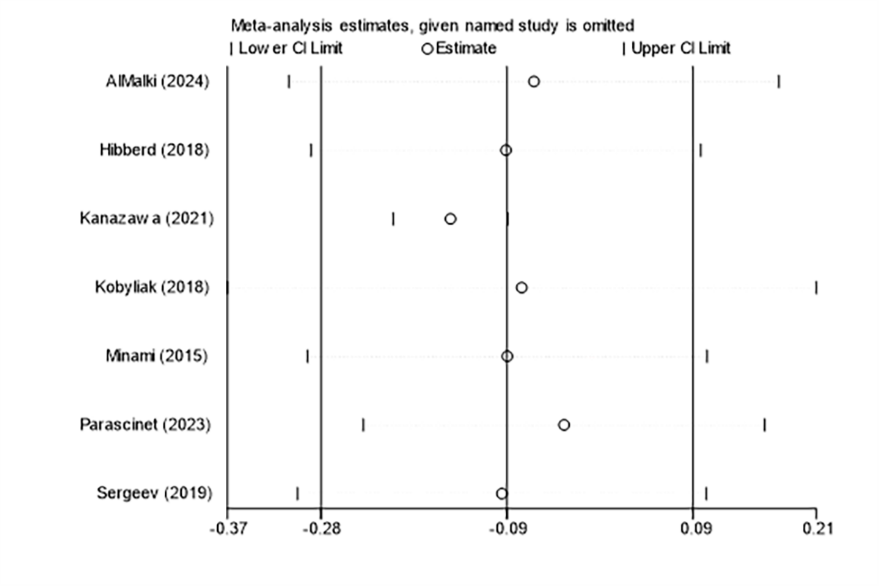


**Supplementary Figure 8** Sensitivity analysis of glycated hemoglobin


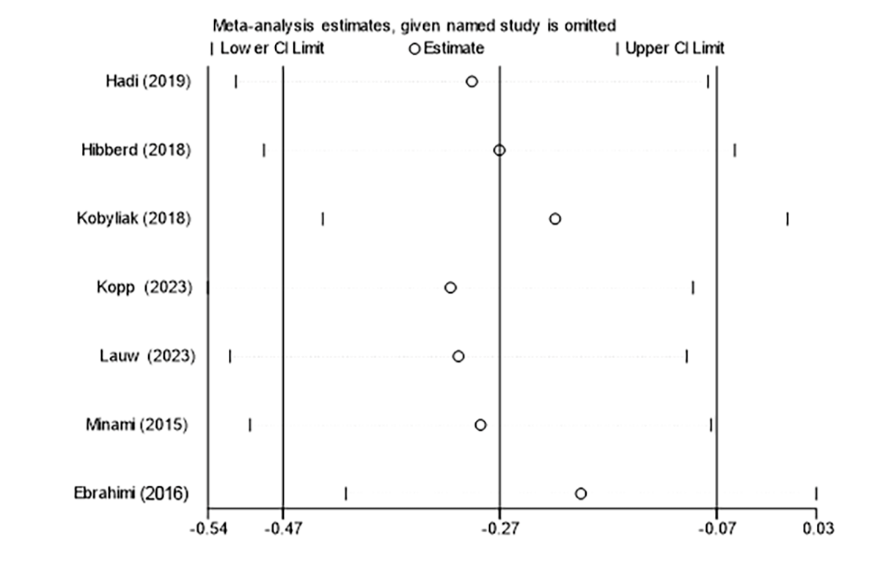


**Supplementary Figure 9** Sensitivity analysis of insulin


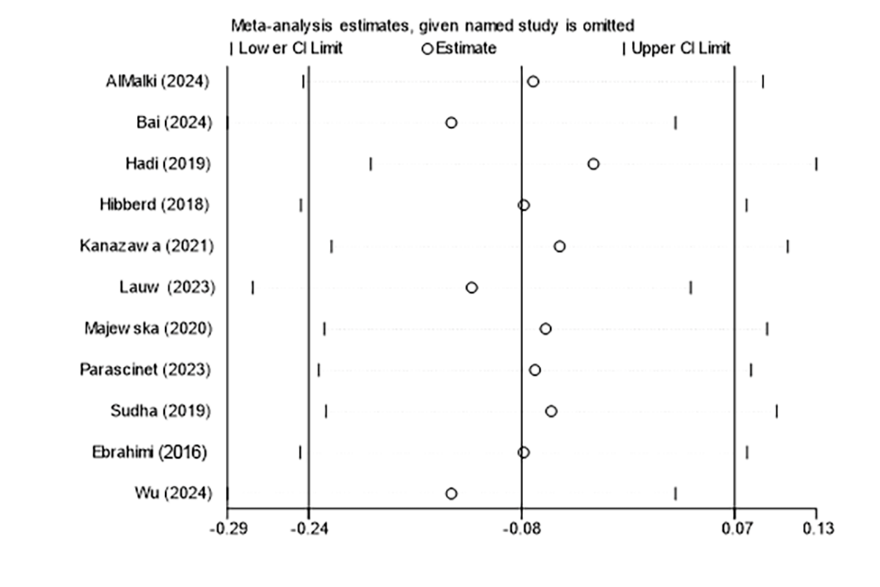


**Supplementary Figure 10** Sensitivity analysis of total Cholesterol


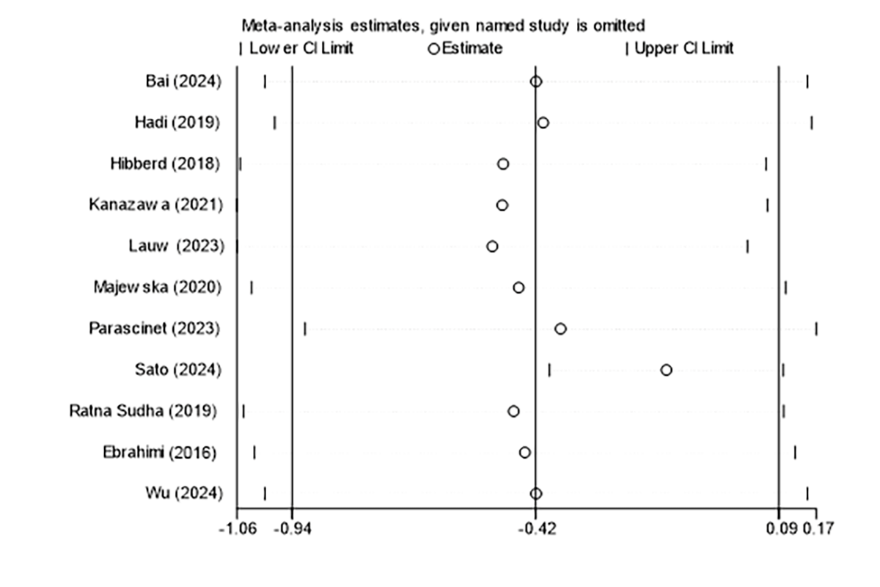


**Supplementary Figure 11** Sensitivity analysis of triglycerides


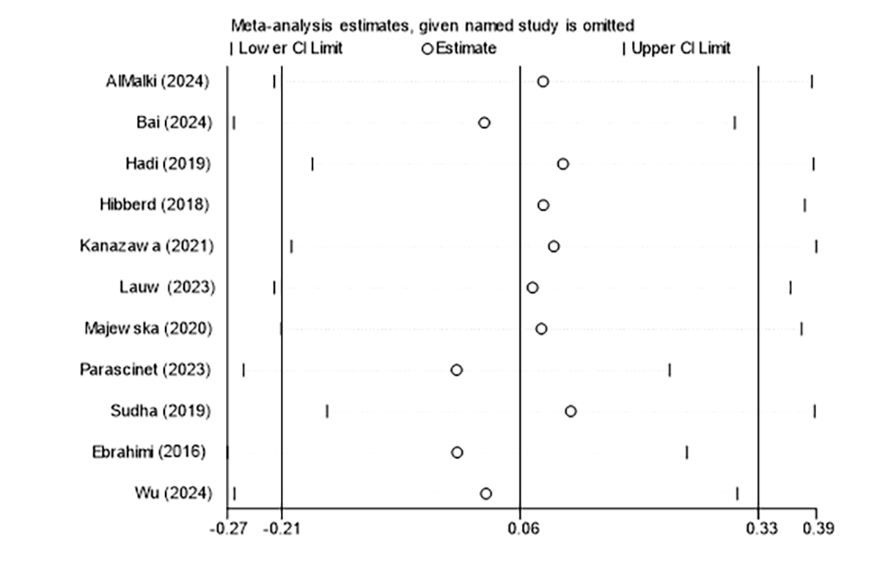


**Supplementary Figure 12** Sensitivity analysis of high-density lipoprotein


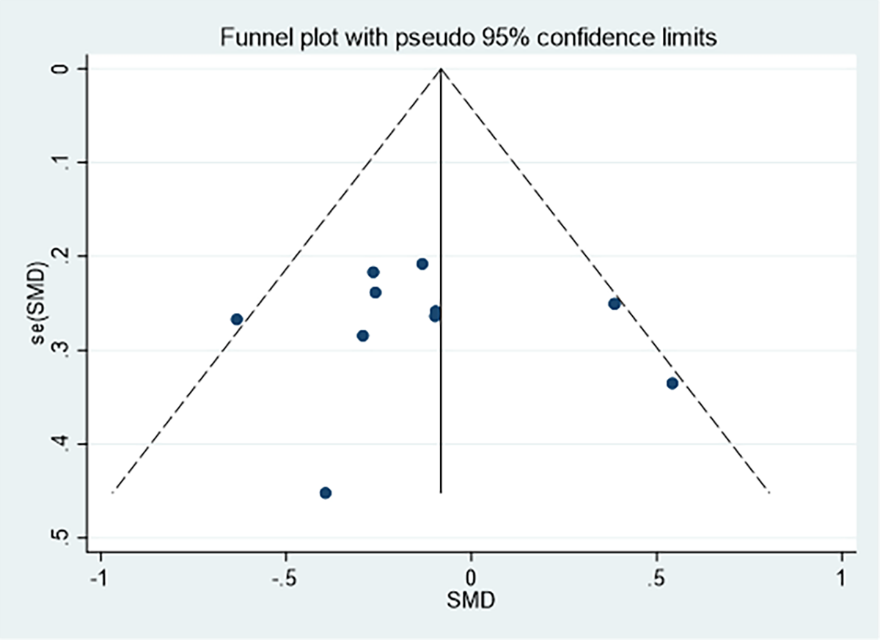


**Supplementary Figure 13** Funnel plot of total cholesterol


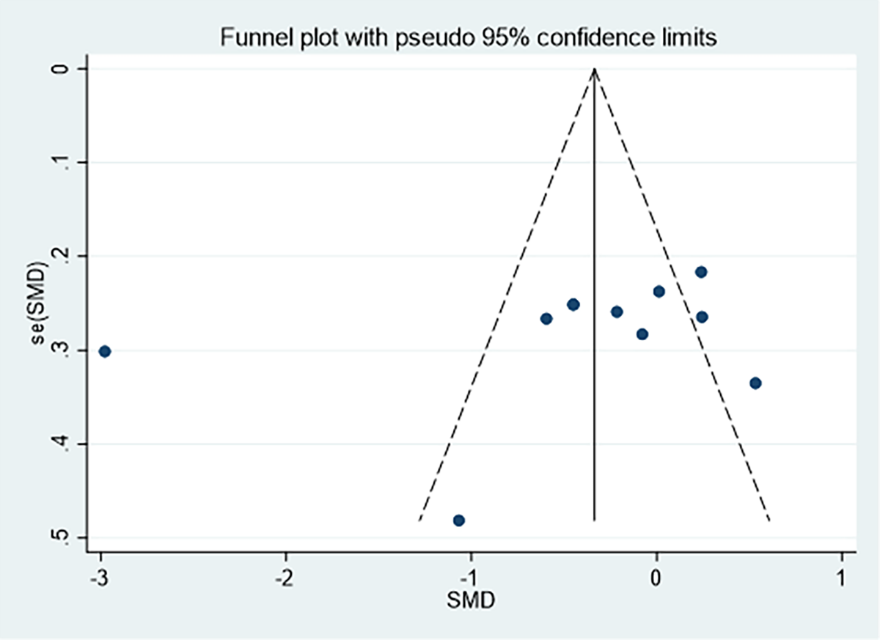


**Supplementary Figure 14** Funnel plot of triglycerides


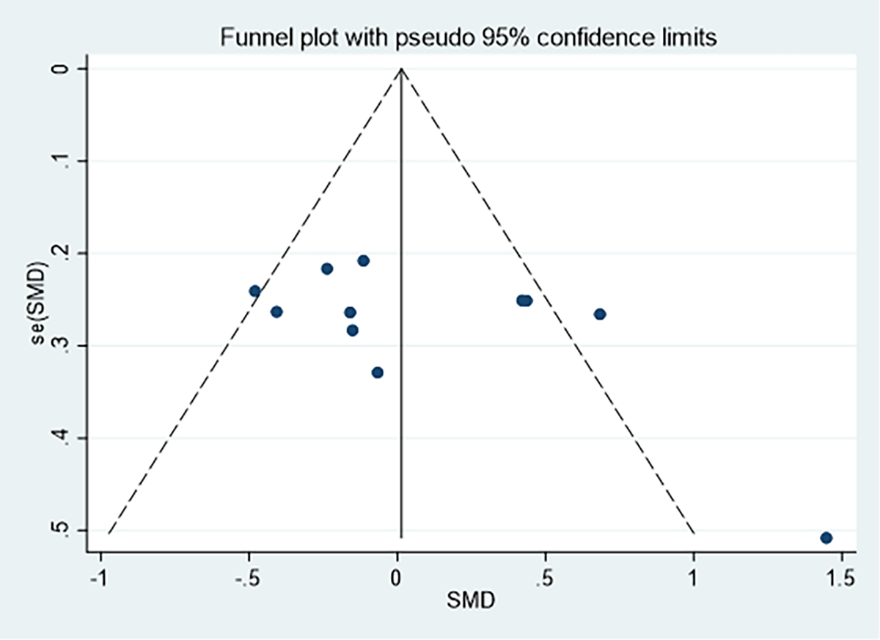


**Supplementary Figure 15** Funnel plot of high-density lipoprotein


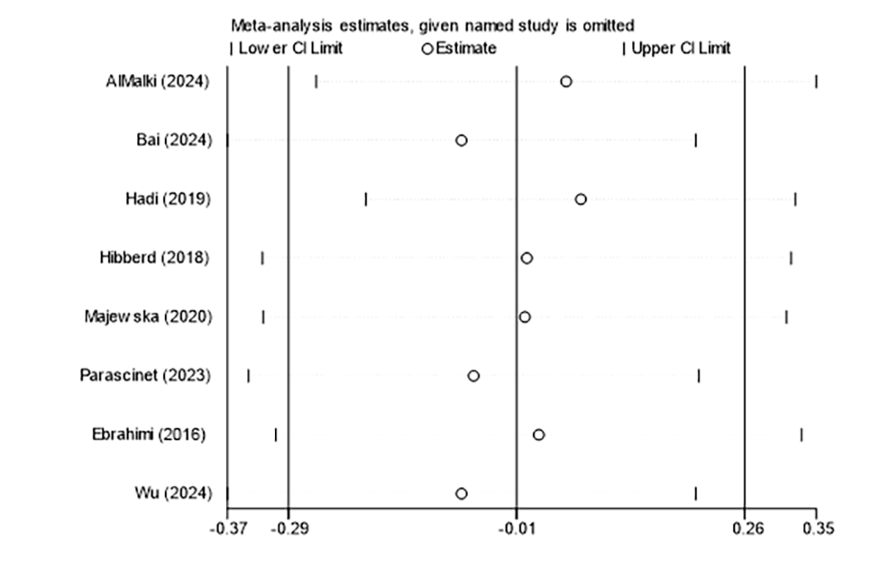


**Supplementary Figure 16** Sensitivity analysis of low-density lipoprotein
